# Socio-demographic and clinical characteristics associated with mental health-related support and service contact in children and young people aged 5–16 in England

**DOI:** 10.1007/s00787-025-02666-0

**Published:** 2025-03-08

**Authors:** Samuel P. Trethewey, Frances Mathews, Abigail Russell, Tamsin Newlove-Delgado

**Affiliations:** Children and Young People’s Mental Health (ChYMe) Research Collaboration, University of Exeter, Exeter, UK

**Keywords:** Mental health, Service contact, Support, Children and young people, Population survey, Inequalities

## Abstract

Mental health problems are common in children and young people (CYP) in England, yet evidence suggests high levels of unmet need. Understanding of the determinants of mental health-related service contact is needed to identify gaps in provision and areas for targeted intervention to improve access. A secondary analysis of the Mental Health of Children and Young People in England 2017 (MHCYP-2017) cross-sectional survey dataset was performed. This analysis describes mental health-related support and service contact amongst a national stratified probability sample of 6681 participants aged 5–16. A range of socio-demographic and clinical characteristics were analysed as explanatory variables and their relationships with different types of support and service contact were examined through multivariable multinomial logistic regression. Analyses were stratified by age group: 5-10- and 11-16-year-olds. Analyses revealed strong associations between participant socio-demographic/clinical characteristics and mental health-related support and service contact, independent of CYP mental health status and parental perception of difficulties. Among these associations, socio-economically disadvantaged and Black, Asian and Minority Ethnic CYP were less likely to have had professional contact for mental health problems in both age groups. Findings suggest there may be higher levels of unmet need in socio-economically disadvantaged and Black, Asian and Minority Ethnic CYP, warranting further investigation and efforts to address inequalities.

**Supplementary Information:**

The online version contains supplementary material available at 10.1007/s00787-025-02666-0.

## Introduction

The mental health of children and young people (CYP) is a public health priority. Recent data suggests that mental disorders are a leading contributor to the global burden of disease among CYP in Europe [1]. In England, the pre-pandemic prevalence of mental health conditions in CYP has increased over time, with the NHS Digital ‘Mental Health of Children and Young People in England 2017’ (MHCYP-2017) survey finding that around 1 in 9 CYP aged 5 to 15 years old in England met the criteria for at least one mental disorder, an increase from 1 in 10 in the previous national survey in 2004 [2, 3].

Crucially, many mental health problems are amenable to evidence-based intervention [4]. However, evidence suggests that even in high-income countries, only a minority of children with disorders are in contact with specialist services. A recent meta-analysis of studies from a diverse range of countries found that only around two fifths of children with a disorder received any services for these conditions [5]. In England, the MHCYP-2017 survey found that less than two thirds of children and young people with a disorder had contact with any professional services in the past year in relation to their mental health [6].

Historically, research across a range of countries and settings has also found that need and access to services are often not evenly matched [7–9]. It is imperative that healthcare providers and policy makers better understand the determinants of mental health-related service contact in CYP, to identify levels of need and areas to focus on improving access to reduce inequalities. However, there is a notable paucity of contemporary research in this area. For example, the most recent nationally representative population-level studies examining predictors of service contact in CYP in the UK include a follow-up sample of 2461 children aged 5–15 from the 1999 British Child and Adolescent Mental Health Survey and a secondary analysis of data from the 2004 Mental Health of Children and Young People in Great Britain Survey [10–11].

This study is the first that we are aware of to investigate the impact of child and family characteristics on mental-health-related support and service contact using data from MHCYP-2017, which is the most recent national survey in England to include clinically validated assessments of psychiatric diagnoses in children. As such, this work builds upon the existing literature by providing insights into specific characteristics associated with different types of mental health-related support and service contact in CYP.

## Methods

### MHCYP-2017

MHCYP-2017 was the third of a series of large-scale national surveys investigating the mental health of CYP, providing the official statistics on CYP mental health in England [12]. Investigators used a stratified multistage random probability sampling approach, drawn from the NHS Patient Register, to recruit a sample of 9,117 CYP aged 2–19 years old [3]. The survey covered a wide range of topics relating to health and wellbeing, including mental health-related contact with a range of informal sources of support and public sector services, as well as socio-demographic characteristics of the CYP and their family.

Data were obtained from multiple informants including the participants themselves (if aged 11 years or older) and/or their parents and teachers. Data were collected through a combination of questionnaires and interviews administered by trained and experienced interviewers. Psychiatric diagnoses were assigned using the Development and Well-Being Assessment (DAWBA), a standardised and validated diagnostic assessment [13]. Expert clinical raters reviewed the DAWBA responses to assign diagnoses, according to established ICD-10 and DSM-V diagnostic criteria [14, 15]. For a full description of the methods of the MHCYP-2017 survey, please see NHS Digital [3].

### Secondary analysis

This study comprises a secondary analysis of CYP aged 5–16 years old in the MHCYP-2017 dataset. Data on CYP outside of this age range were not considered in this secondary analysis due to key differences in data collection methods employed in the 2–4 and 17–19 age groups. We used the data obtained from the ‘main informant’, which was the parent/guardian (from here onwards, referred to as ‘parent’).

### Support and service contact

Data on parent reported mental health-related support and service contact in the past 12 months was available for 6681 participants aged 5–16 years old. Parents were asked if they had been in contact with one or more of a range of types of support/services during the last year, due to concerns regarding CYP “emotions, behaviour, concentration or difficulties in getting along with people”. The potential response categories were a mixture of informal support and professional services (see Supplementary Information 1).

Several binary service contact variables were generated by coding the original survey responses to facilitate the current analysis, organising responses into three outcome groups: ‘informal contact’, ‘professional contact’ and ‘specialist mental health contact’ (see Table 1). A combined multilevel variable was created to summarise service contact across four levels of outcome: (1) no contact, (2) informal contact, (3) professional contact, (4) specialist mental health contact. For participants with multiple levels of contact, only the highest level was coded within the combined variable.

**Table 1:** Support and service contact variables

Informal contact	Someone in your family or a close friend
	Telephone help line
	Self-help group
	Internet
Professional contact	A teacher (including form tutor, head of year, head teacher or coordinator)
	Someone working in additional support services (for example an educational psychologist, educational social worker or specialist teacher from outside school)
	Someone from primary health care such as your GP, family doctor, health visitor, practice nurse or school nurse
	Someone specialising in children’s physical health, such as a hospital or community paediatrician, or occupational therapist
	Someone from social care, such as a social worker
	Someone from youth justice, such as a probation officer or someone working in a Youth Offending Team
Specialist mental health contact	Someone specialising in mental health care, such as a mental health nurse, psychiatrist, psychologist or counsellor

### Explanatory variables

A range of socio-demographic and clinical characteristics of the CYP and family were analysed as explanatory variables including: age, sex, ethnicity, region, physical health status (presence or absence of any physical disorder), mental health status (presence or absence of one or more mental health disorders determined via the DAWBA) [13], history of stressful life events (SLEs: for example, parental separation, financial crisis, involvement with police, parental or sibling death), special educational needs (SEN), history of exclusion or managed moves between primary schools, CYP caring responsibilities, parent marital status, indicators of socio-economic status at both the household (tenure of household) and neighbourhood level (Index of Multiple Deprivation: IMD), parent mental health (measured by the 12-item General Health Questionnaire: GHQ-12) [16], family functioning (measured by General Functioning Scale of the McMaster Family Activity Device: FAD) [17] and total difficulties score (measured by the parent version of the Strength and Difficulties Questionnaire: SDQ) [18].

Variable selection was theoretically informed in that potential determinants of service contact were identified through literature review and final decisions on variable inclusion were achieved iteratively through discussion and consensus within the study team (see Supplementary Information 6 for a summary of the evidence base considered). To assess completeness of the dataset, a summary table was constructed showing the frequencies of missing data for each explanatory variable (Supplementary Information 2). As the proportion of missing data was < 2% for all included variables, no adjustments were necessary for missing data.

### Statistical analysis

Analyses were conducted in Stata SE version 17.0 (StataCorp LLC, College Station, Texas, USA). Descriptive statistics were used to summarise participant characteristics of the whole sample and according to different types of support and service contact. Categorical variables were presented as number (n) and percentage (%); continuous data were presented as mean and standard deviation (SD). In reporting raw numbers, the ONS and UK Data Service Statistical Disclosure Controls guidance was followed according to our Data Sharing Agreement with NHS Digital.

Multivariable multinomial logistic regression was used to determine the relationships between each of the explanatory variables and the multilevel outcome comprising: (1) no contact, (2) informal contact, (3) professional contact, (4) specialist mental health contact. In all models, ‘no contact’ was taken as the base outcome. Outputs are presented as odds ratios (ORs) and associated 95% confidence intervals and p-values. Separate ORs are produced for all three comparison outcome groups (informal contact, professional contact and specialist mental health contact) with reference to the base outcome group (no contact). Additional explanation of the multivariable model building process can be found in Supplementary Information 3.

Analyses were stratified by age group, such that data are presented separately for 5-10- and 11-16-year-olds. This stratification intentionally separates primary and secondary school age groups, as there may be considerable differences in access to different types of mental health support. Moreover, this stratification may help to produce more granular findings which may be interpreted more easily by practitioners and policy makers. Due to small numbers of individual ethnic groups in stratified analyses, a dichotomous ethnicity variable was used comprising: (1) Black (Black/African/Caribbean/Black British), Asian (Asian/Asian British) and Minority (Mixed/Multiple ethnic groups/Other) Ethnic (BAME) and (2) White (White British/White other) ethnic groups.

## Results

In total, 1656 participants (24.8%) reported contact with one or more types of support/service in the past 12 months. When stratified by the highest reported level of support/service contact, 255 (3.8%) reported ‘informal contact’, 1139 (17.0%) reported ‘professional contact’ and 262 (3.9%) reported ‘specialist mental health contact’. Table 2 summarises participant characteristics for the whole study sample and stratified by the highest level of support/service contact reported.

**Table 2:** Participant characteristics

Variable	Whole sample (*n* = 6681)	Stratified by highest level of support/service contact			
		No contact (*n* = 5025)	Informal contact (*n* = 255)	Professional contact (*n* = 1139)	Specialist mental health contact (*n* = 262)
**Age (years) - mean (SD)**	10.2 (3.4)	10.1 (3.5)	10.6 (3.5)	10.1 (3.3)	11.6 (3.0)
**Sex**					
Male	3351 (50.2%)	2455 (48.9%)	124 (48.6%)	630 (55.3%)	142 (54.2%)
Female	3330 (49.8%)	2570 (51.1%)	131 (51.4%)	509 (44.7%)	120 (45.8%)
**Ethnicity**					
White	5299 (79.3%)	3825 (76.2%)	219 (85.9%)	1015 (89.1%)	240 (91.6%)
BAME	1380 (20.7%)	1198 (23.9%)	36 (14.1%)	124 (10.9%)	22 (8.4%)
**Region** London and South of	2470 (37.0%)	1873 (37.3%)	96 (37.7%)	415 (36.4%)	86 (32.8%)
England					
North of England	2106 (31.5%)	1579 (31.4%)	79 (31.0%)	356 (31.3%)	92 (35.1%)
Midlands and East of England	2105 (31.5%)	1573 (31.3%)	80 (31.4%)	368 (32.3%)	84 (32.1%)
**Any physical disorder**					
No	3254 (48.7%)	2687 (53.5%)	105 (41.2%)	378 (33.2%)	84 (32.1%)
Yes	3427 (51.3%)	2338 (46.5%)	150 (58.8%)	761 (66.8%)	178 (67.9%)
Any mental health disorder*					
0	5760 (86.2%)	4748 (94.5%)	205 (80.4%)	731 (64.2%)	76 (29.0%)
1	589 (8.8%)	239 (4.8%)	36 (14.1%)	241 (21.2%)	73 (27.9%)
≥ 2	332 (5.0%)	38 (0.8%)	14 (5.5%)	167 (14.7%)	113 (43.1%)
**Two or more stressful life events**					
No	5390 (80.9%)	4272 (85.2%)	180 (70.6%)	783 (68.9%)	155 (59.9%)
Yes	1272 (19.1%)	740 (14.8%)	75 (29.4%)	353 (31.1%)	104 (40.2%)
**Special educational needs**					
No	6099 (91.6%)	4823 (96.1%)	236 (92.6%)	882 (78.0%)	158 (61.5%)
Yes	563 (8.5%)	196 (3.9%)	19 (7.5%)	249 (22.0%)	99 (38.5%)
**Ever excluded or had a managed move between primary schools**					
No	6427 (97.2%)	4915 (98.7%)	246 (96.5%)	1050 (93.2%)	216 (87.5%)
Yes	183 (2.8%)	66 (1.3%)	10 (3.5%)	77 (6.8%)	31 (12.6%)
**CYP has caring responsibilities**					
No	5984 (89.7%)	4513 (89.9%)	222 (87.1%)	1018 (89.5%)	231 (88.9%)
Yes	687 (10.3%)	506 (10.1%)	33 (12.9%)	119 (10.5%)	29 (11.2%)
**Tenure of household**					
Owner occupied	4054 (60.8%)	3123 (62.2%)	150 (59.3%)	635 (55.8%)	146 (55.7%)
Private rented	1233 (18.5%)	900 (17.9%)	61 (24.1%)	224 (19.7%)	48 (18.3%)
Social rented	1385 (20.8%)	996 (19.8%)	42 (16.6%)	279 (24.5%)	68 (26.0%)
**Neighbourhood deprivation by IMD**					
Least deprived to 3rd quintile	3737 (55.9%)	2723 (54.2%)	160 (62.8%)	696 (61.1%)	158 (60.3%)
4th quintile to most deprived	2944 (44.1%)	2302 (45.8%)	95 (37.3%)	443 (38.9%)	104 (39.7%)
**HRP marital status**					
Married or cohabiting	5222 (78.2%)	4054 (80.7%)	187 (73.3%)	816 (71.6%)	165 (63.0%)
Lone parent	1459 (21.8%)	971 (19.3%)	68 (26.7%)	323 (28.4%)	97 (37.0%)
**Parent mental health score by GHQ-12**					
0–3 (good)	5617 (85.0%)	4380 (88.0%)	210 (83.3%)	858 (76.5%)	169 (66.0%)
≥ 4 (poor)	989 (15.0%)	597 (12.0%)	42 (16.7%)	263 (23.5%)	87 (34.0%)
**Family functioning score by McMaster FAD**					
≤ 2 (healthy)	5492 (83.5%)	4244 (85.6%)	192 (76.2%)	874 (78.3%)	182 (71.7%)
> 2 (unhealthy)	1087 (16.5%)	712 (14.4%)	60 (23.8%)	243 (21.8%)	72 (28.4%)
Total difficulties score on parent SDQ** - mean (SD)	8.0 (6.2)	6.4 (4.7)	9.6 (6.2)	12.5 (7.1)	18.1 (7.9)

Abbreviations: BAME, Black, Asian and Minority Ethnic; CYP, children or young person; IMD, Index of Multiple Deprivation; HRP, household reference person; GHQ-12, 12-item General Health Questionnaire; FAD, Family Assessment Device; SDQ, Strengths and Difficulties Questionnaire. NB: summary statistics are reported to 1 decimal place. *Presence of mental health disorder determined via the DAWBA. **Score ranges from 0–40, with a higher score indicating greater perceived difficulties

### Multinomial logistic regression

Fully adjusted multivariable models showing explanatory variables with statistically significant associations with one or more types of support/service contact are reported here. Supplementary univariable analyses with accompanying commentary can be found in Supplementary Information 4 and 5.

### 5–10-year-Olds

Table 3 summarises the output from the final, fully adjusted multivariable model for 5–10-year-olds (*n* = 3489). Age, ethnicity, physical disorder status, mental health disorder status, SLEs, SEN, household tenure, neighbourhood deprivation, parent mental health status and total difficulties score all demonstrated statistically significant associations with one or more types of support/service contact. The strength of evidence for these associations ranged from very strong (*p* < 0.001) to moderate (*p* < 0.05). The strongest association for specialist mental health contact was having two or more mental health disorders (OR 12.17; 95% CI 4.90-30.24, *p* < 0.001). BAME ethnicity was associated with a lower odds of contact compared to White ethnicity for professional contact (OR 0.47; 95% CI 0.35–0.63, *p* < 0.001). Living in socially rented accommodation (versus living in owner occupied accommodation) or living in an area of relative deprivation (as measured by the IMD) were also both independently associated with lower odds of contact across all three support/service categories.

**Table 3:** Fully adjusted multivariable multinomial logistic regression model for 5-10-year-olds

Variable	Informal contact			Professional contact			Specialist mental health contact		
	OR	95% CI	*P* value	OR	95% CI	*P* value	OR	95% CI	*P* value
**Age (years)**	1.07	0.96–1.20	0.205	**1.08**	**1.02–1.15**	**0.011**	**1.33**	**1.14–1.55**	**< 0.001**
**Ethnicity**									
White (ref.)	-	-	-	**-**	**-**	**-**	-	-	-
BAME	0.64	0.38–1.07	0.089	**0.47**	**0.35–0.63**	**< 0.001**	0.46	0.20–1.05	0.066
**Any physical disorder**									
No (ref.)	-	-	-	**-**	**-**	**-**	-	-	-
Yes	1.41	0.97–2.06	0.073	**1.38**	**1.12–1.69**	**0.002**	1.39	0.81–2.39	0.238
**Any mental health disorder**									
0 (ref.)	**-**	**-**	**-**	**-**	**-**	**-**	**-**	**-**	**-**
1	**1.94**	**1.08–3.51**	**0.028**	**2.60**	**1.89–3.58**	**< 0.001**	**3.28**	**1.62–6.63**	**0.001**
≥ 2	0.89	0.19–4.30	0.887	**4.17**	**2.13–8.17**	**< 0.001**	**12.17**	**4.90-30.24**	**< 0.001**
**Two or more stressful life events**									
No (ref.)	-	-	-	**-**	**-**	**-**	**-**	**-**	**-**
Yes	1.59	0.97–2.60	0.063	**1.79**	**1.38–2.32**	**< 0.001**	**1.84**	**1.02–3.33**	**0.043**
**Special educational needs**									
No (ref.)	-	-	-	**-**	**-**	**-**	**-**	**-**	**-**
Yes	1.11	0.50–2.43	0.798	**3.10**	**2.19–4.39**	**< 0.001**	**3.73**	**2.04–6.85**	**< 0.001**
**Tenure of household**									
Owner occupied (ref.)	-	-	-	-	-	-	-	-	-
Private rented	1.23	0.78–1.92	0.370	1.00	0.77–1.29	0.975	0.91	0.48–1.75	0.780
Social rented	**0.54**	**0.31–0.95**	**0.033**	**0.69**	**0.52–0.91**	**0.009**	**0.49**	**0.25–0.95**	**0.035**
**Neighbourhood deprivation by IMD**									
Least deprived to 3rd quintile (ref.)	**-**	**-**	**-**	**-**	**-**	**-**	**-**	**-**	**-**
4th quintile to most deprived	**0.58**	**0.39–0.86**	**0.007**	**0.61**	**0.49–0.75**	**< 0.001**	**0.54**	**0.32–0.90**	**0.017**
**Parent mental health score by GHQ-12**									
0–3 (ref.)	-	-	-	**-**	**-**	**-**	-	-	-
≥ 4	0.98	0.57–1.67	0.928	**1.39**	**1.06–1.82**	**0.017**	1.06	0.58–1.92	0.852
**Total difficulties score on parent SDQ**	**1.12**	**1.08–1.16**	**< 0.001**	**1.13**	**1.11–1.15**	**< 0.001**	**1.23**	**1.18–1.29**	**< 0.001**

Abbreviations: BAME, Black, Asian and Minority Ethnic; IMD, Index of Multiple Deprivation; GHQ-12, 12-item General Health Questionnaire; SDQ, Strengths and Difficulties Questionnaire; ref., reference category; OR, Odds Ratio; 95% CI, 95% confidence interval. NB: OR and 95% CI are reported to two decimal places. In all models, ‘no contact’ is the base outcome

### 11–16-year-olds

Table 4 summarises the output from the final, fully adjusted multivariable model for 11-16-year-olds (*n* = 2968). Age, ethnicity, physical disorder status, mental health disorder status, SLEs, SEN, exclusion from school, household tenure, neighbourhood deprivation, marital status of the household reference person (HRP) and total difficulties score all demonstrated statistically significant associations with one or more types of support/service contact. Similar to the 5–10 age group, the strongest association for specialist mental health contact was having two or more mental health disorders (OR 17.87; 95% CI 8.76–36.44, *p* < 0.001). BAME ethnicity was also associated with a lower odds of contact compared to White ethnicity for professional contact (OR 0.56; 95% CI 0.39–0.79, *p* = 0.001).

**Table 4:** Fully adjusted multivariable multinomial logistic regression model for 11-16-year-olds

Variable	Informal contact			Professional contact			Specialist mental health contact		
	OR	95% CI	*P* value	OR	95% CI	*P* value	OR	95% CI	*P* value
**Age (years)**	1.07	0.96–1.20	0.213	**0.92**	**0.86–0.99**	**0.022**	0.98	0.87–1.10	0.692
**Ethnicity**									
White (ref.)	-	-	-	**-**	**-**	**-**	-	-	-
BAME	0.72	0.42–1.23	0.233	**0.56**	**0.39–0.79**	**0.001**	0.53	0.26–1.07	0.075
**Any physical disorder**									
No (ref.)	-	-	-	**-**	**-**	**-**	-	-	-
Yes	1.25	0.85–1.84	0.248	**1.60**	**1.26–2.03**	**< 0.001**	0.82	0.54–1.25	0.360
**Any mental health disorder**									
0 (ref.)	**-**	**-**	**-**	**-**	**-**	**-**	**-**	**-**	**-**
1	**2.24**	**1.25–4.02**	**0.007**	**3.05**	**2.17–4.28**	**< 0.001**	**7.64**	**4.53–12.89**	**< 0.001**
≥ 2	**4.11**	**1.73–9.73**	**0.001**	**5.17**	**2.97–9.02**	**< 0.001**	**17.87**	**8.76–36.44**	**< 0.001**
**Two or more stressful life events**									
No (ref.)	**-**	**-**	**-**	**-**	**-**	**-**	**-**	**-**	**-**
Yes	**2.03**	**1.34–3.08**	**0.001**	**1.99**	**1.53–2.58**	**< 0.001**	**2.18**	**1.41–3.36**	**< 0.001**
**Special educational needs**									
No (ref.)	-	-	-	**-**	**-**	**-**	-	-	-
Yes	0.75	0.36–1.56	0.443	**1.49**	**1.03–2.17**	**0.035**	1.53	0.90–2.60	0.120
**Ever excluded or had a managed move between primary schools**									
No (ref.)	-	-	-	**-**	**-**	**-**	-	-	-
Yes	1.72	0.78–3.80	0.179	**2.03**	**1.22–3.36**	**0.006**	1.10	0.54–2.24	0.802
**Tenure of household**									
Owner occupied (ref.)	-	-	-	-	-	-	**-**	**-**	**-**
Private rented	1.33	0.82–2.16	0.254	0.94	0.68–1.32	0.730	**0.46**	**0.25–0.86**	**0.014**
Social rented	0.71	0.41–1.23	0.222	0.83	0.60–1.15	0.259	**0.45**	**0.26–0.77**	**0.003**
**Neighbourhood deprivation by IMD**									
Least deprived to 3rd quintile (ref.)	-	-	-	**-**	**-**	**-**	-	-	-
4th quintile to most deprived	0.71	0.48–1.05	0.090	**0.67**	**0.52–0.86**	**0.001**	0.72	0.48–1.10	0.130
**HRP marital status**									
Married or cohabiting (ref.)	**-**	**-**	**-**	-	-	-	**-**	**-**	**-**
Lone parent	**1.57**	**1.02–2.43**	**0.042**	1.08	0.81–1.44	0.582	**1.71**	**1.09–2.70**	**0.021**
**Total difficulties score on parent SDQ**	**1.08**	**1.04–1.12**	**< 0.001**	**1.12**	**1.10–1.15**	**< 0.001**	**1.18**	**1.14–1.23**	**< 0.001**

Abbreviations: BAME, Black, Asian and Minority Ethnic; IMD, Index of Multiple Deprivation; HRP, household reference person; SDQ, Strengths and Difficulties Questionnaire; ref., reference category; OR, Odds Ratio; 95% CI, 95% confidence interval. NB: OR and 95% CI are reported to two decimal places. In all models, ‘no contact’ is the base outcome

## Discussion

In this national probability sample of CYP aged 5–16 years old living in England, around a quarter of parents reported contact with one or more types of support/service in the past 12 months due to concerns regarding CYP “emotions, behaviour, concentration or difficulties in getting along with people”. Analyses revealed several important associations between participant socio-demographic/clinical characteristics and parent-reported mental health-related support and service contact. These associations were not necessarily consistent across outcome categories, suggesting that several of the measured characteristics have differential relationships with different levels of support and service contact as categorised in this study.

### Socio-demographics

Increased age was associated with a higher odds of professional contact and specialist mental health contact in the 5–10 age group. The observed positive associations may partly reflect a time-lag to accessing support for younger children due to lower parental recognition of needs, parents adopting a ‘wait and see’ approach, long wait lists for specialist assessment and/or barriers to accessing services [19, 20]. Lone parents (single or previously married) were more likely to report informal or professional contact when compared to parents who were married or cohabiting. This finding is in-keeping with a systematic review which found that CYP with mental health problems from lone-parent households had a higher odds of mental health service use, which may reflect lower availability of psychosocial resources from lone parents alongside a lack of partner support from within the household, manifesting in increased external help-seeking [21].

Ethnicity showed a strong and consistent relationship with support/service contact in both age groups, with BAME participants being significantly less likely than White ethnicity participants to have had professional contact relative to no contact in adjusted analyses. These findings may therefore indicate inequality of access to professional services, and higher levels of unmet need in BAME participants, which may partly reflect wider issues regarding ethnic inequalities and barriers to accessing mental health support [22]. A recent systematic scoping review identified several barriers to accessing services for CYP from minority ethnic backgrounds, including cultural differences in understanding of and attitudes towards mental health problems and services, stigma, and a lack of trust in the healthcare system [23].

Socio-economic status, as measured at both the household and neighbourhood levels, was associated with support/service contact in both age groups. In terms of accommodation status, children and young people residing in social rented accommodation had a lower odds of specialist service contact. These families may be relatively socio-economically disadvantaged and thus may plausibly experience financial barriers to accessing mental health care including the direct cost of travelling to appointments and the indirect opportunity cost of lost working hours [24, 25].

At the neighbourhood level, participants living in more deprived neighbourhoods were also less likely to have had contact with a professional service. Whilst the mechanisms underlying these findings are unclear, provision of mental health services may be disproportionately lower in deprived areas and recognition of mental health needs may be impaired in under-resourced schools [26–28]. In addition, lower health literacy and lower perceived severity of problems relative to others living in low socio-economic status areas may also be contributing to lower rates of service contact [27, 29].

A history of two or more SLEs was strongly associated with increased odds of support/service contact in both age groups. This finding is partly in line with a previous analysis of follow-up data from an earlier national survey, which found that three or more SLEs was associated with increased odds of contact with mental health services in older children [10]. It is also possible that CYP with a history of multiple SLEs may be more likely to encounter professionals and services for reasons other than mental health, for example children’s social services, and that this might then facilitate greater recognition of mental health needs and access to support.

### Clinical characteristics

Having a physical health condition was associated with an increased odds of professional contact in both age groups. This finding may reflect that the professional contact category includes primary care professionals and children’s physical health professionals, whom may already be supporting the CYP with their physical condition and thus may also be alert to/available for discussion of mental health concerns. Alternatively, this finding may indicate increased mental health needs in CYP with physical conditions. Evidence from a recent systematic scoping review indicates a high prevalence of comorbid mental disorders in CYP with physical illnesses, and that physical-mental comorbidity has measurable impacts on well-being and quality of life [30].

Participants who met the criteria for a mental health disorder had a significantly higher odds of all types of support/service contact in both age groups, with the strongest associations seen with specialist mental health contact. Moreover, participants with ≥ 2 mental health disorders had even greater odds of contact, again most notably with regards to specialist mental health services in both the 5–10 (OR 12.17, *p* < 0.001) and 11–16 (OR 17.87, *p* < 0.001) age groups. Total difficulties score on parent SDQ was also strongly and consistently (*P* < 0.001 for all comparisons) associated with all types of support/service contact in both age groups. These findings are in-keeping with previous analyses of UK-based data, which highlights the role of parental recognition of CYP difficulties via the SDQ in predicting mental-health related service use [29, 31].

Finally, parent mental health was associated with professional contact in the 5–10 age group. There are complex bi-directional interactions between parent and child mental health and the wider environmental contexts in which families live, such that pathways to accessing support/service contact are themselves highly complex [32]. Whilst we cannot infer causality from this data, it may be the case that parents with increased mental health difficulties may be more likely to require additional external support when managing CYP with mental health problems. Conversely, parents who are already in contact with services for their own mental health may experience fewer barriers to referral or support for their child, in contrast to families unknown to services.

### Strengths and limitations

A key strength of this study is the use of a large, national stratified probability sample. MHCYP-2017 also used a multi-informant approach with complementary data collection methods using validated tools. Another key strength of this dataset is the meticulous process that was used to identify the presence of mental disorders through expert clinical rating of individual participant data obtained via the DAWBA. Moreover, information was collected on parent-reported contact with a wide range of support and service categories. As a result, the data collection methods utilised in MHCYP-2017 enabled detailed analyses of the relationships between both socio-demographics and clinical characteristics with support and service contact.

This study is subject to several methodological limitations. Service contact was determined through yes or no responses to a multiple answer question. We therefore do not know precisely when this contact took place, nor do we know the exact purpose or frequency of contact so cannot differentiate between participants reporting a single contact versus multiple contacts with the same type of support/service. The multilevel outcome variable used in this study encapsulates a range of different types of support/services (informal all the way to specialist mental health) which supplements previous work in the field which largely focusses on only professional and specialist services. Notably, however, the ‘professional’ (non-mental health specialist) outcome level groups several different types of professionals together, who may themselves play very different roles in supporting CYP and thus may have differential relationships with the explanatory variables. However, due to small numbers reporting contact with several types of professional, there was not sufficient statistical power to further separate out these categories and increase the number of levels of the multilevel outcome. Further work is needed to explore the relationships between socio-demographic/clinical characteristics and specific types of professional services.

Due to small numbers of individual ethnic groups in stratified analyses, a dichotomous ethnicity variable was used comprising BAME and White ethnic groups. However, we recognise that there might be further variation in associations with service contact across minoritised ethnic groups as BAME is not a homogenous construct; this needs further exploration in studies with more diverse samples. We cannot infer causality from cross-sectional data, however given the temporality of the relationships between key socio-demographic variables and the outcome of interest, some direction of association can be inferred.

In some cases, contact with services may have been by the parent only, although this will have been due to concerns about the CYP. Finally, determination of service contact relies on accurate recall and parents may not always be made aware of all instances of service contact that the CYP received, particularly with older children and informal support, which may lead to under-reporting. However, previous validation studies have shown that the Childrens Services Interview utilised in this study demonstrates moderate or better concordance with medical records about appointments and interventions, although agreement is poorer about the type of professional seen [33].

### Policy and practice implications and recommendations for further research

This study builds upon the existing literature by providing insights into characteristics associated with different types of mental health-related support and service contact in CYP. The finding that socio-economically disadvantaged and BAME CYP were less likely to report professional contact for mental health problems warrants further investigation as this may represent inequalities in access to services and unmet need in these populations. If the observed results do indeed represent unmet need in these population groups, further work is needed to explore the mechanisms driving these observations and possible barriers to accessing support. A range of possible factors may be implicated, ranging from availability and accessibility of services through to acceptability, including issues such as higher rates of mental health stigma.

Concerningly, recent findings suggest that that the prevalence of mental health problems in CYP in England has risen further during the pandemic [34] which may place additional pressure on already struggling mental health services and may widen existing inequalities [35]. These findings are therefore of importance for policymakers and the broad range of professionals supporting CYP across the life course, to inform regional and local health needs assessments, identify gaps in service provision and guide resource allocation to improve access for under-served groups.

Crucially, there are many potential upstream factors which can influence the complex pathway from parental recognition of CYP mental health problems to appropriate mental health service use [36]. Many of these aspects could not be accounted for in this study, such that important associations may have been missed. For example, further research is needed to investigate the role of other potential determinants of service use including aspects of parent-CYP relationships, mental health literacy, perceptions of mental health problems, social capital and attitudes towards mental health services. Structural and service-level factors such as distance from services, caseloads and availability of specialists are also important determinants of service use and should be considered in future analyses.

Given the complexity of assessing socio-economic status, particularly in the context of composite neighbourhood-level measures such as IMD, there is a need for further studies to investigate how specific aspects of deprivation and disadvantage influence access to support and services. Further research is also needed to better understand the cumulative impact of multiple types of adversity on service contact. More detailed analyses of regional and local data are also needed to identify specific areas where access to mental health support is poorer and to help address inequalities.

Further, prospective studies are needed to better describe the nature of service contact, with more precision around timing and frequency of contacts, to better understand different patterns of support and pathways through services. This would also help us to better understand how patterns of service use change for individuals throughout the transition from childhood through to adolescence and beyond. Longitudinal follow-up of participants may also help to reduce recall bias and better understand outcomes following contact with different types of support and services. Analyses of future waves of national mental health survey data should also seek to explore how predictors of service contact vary according to different types of underlying mental health problem and different categories of special educational needs.

## Conclusion

In this national stratified probability sample of 6681 CYP aged 5–16 years old living in England, around a quarter of parents reported mental health-related contact with one or more types of support/service in the past 12 months. Analyses revealed several important associations between socio-demographic/clinical characteristics and mental health-related support and service contact, independent of CYP mental health status and parental perception of difficulties. These findings highlight the complexity of mental health-related service contact in CYP, with differential relationships observed according to different types of support and between age groups. Notwithstanding the limitations of the cross-sectional study design, the findings suggest there may be higher levels of unmet need in some groups, warranting further investigation and efforts to address inequalities. Further longitudinal studies are needed to elucidate causal associations and mechanisms underlying these observations.

## Electronic supplementary material

Below is the link to the electronic supplementary material.

Supplementary Material 1

## Data Availability

The MHCYP 2017 dataset is archived with the UK Data Service. Approval for access to the MHYCP data can be obtained through the UK Data Service Data Access Request Service.
