# Supplementary material for: Socio-demographic and clinical characteristics associated with mental health-related support and service contact in children and young people aged 5–16 in England
Source: Eur Child Adolesc Psychiatry. 2025 Mar 8;34(8):2599–608. doi: 10.1007/s00787-025-02666-0 (PMC12397150; doi:10.1007/s00787-025-02666-0)
Supplement: Supplementary file 1 — Supplementary Material 1 [file 787_2025_2666_MOESM1_ESM.pdf]

Article title: Socio-demographic and clinical characteristics associated with mental health-related support and service contact in children and young people aged 5-16 in England.

Authors: Samuel P. Trethewey[1], Frances Mathews[1], Abigail Russell[1], Tamsin Newlove-Delgado[1]

Affiliations: 1. Children and Young People's Mental Health (ChYMe) research collaboration, University of Exeter, Exeter, UK.

Corresponding author email: s-trethewey@doctors.org.uk

Supplementary Information 1. Support and service contact questions.

The following extract of the service contact question is taken from the MHCYP-2017 survey resources which can be found on the NHS Digital website (NHS Digital. *Mental Health of Children and Young People in England, 2017 Appendix B: Questionnaire.* Available at: https://files.digital.nhs.uk/D7/BDF0AD/MHCYP%202017%20Appendix%20B%20-%20Questionnaire.pdf)

"Here is a list of people who parents and young people often turn to when they want advice and treatment about a young person's emotions, behaviour, concentration or difficulties in getting along with people. In the past year have you or <Name> been in contact with any of these people because of worries about his/her emotions, behaviour, concentration or difficulties in getting along with people?

1. Someone in your family or a close friend
2. Telephone help line
3. Self-help group
4. Internet
5. A teacher (including form tutor, head of year, head teacher or coordinator)?
6. Someone working in additional support services (for example an educational psychologist, educational social worker or specialist teacher from outside school)
7. Someone from primary health care such as your GP, family doctor, health visitor, practice nurse or school nurse
8. Someone specialising in mental health care, such as a mental health nurse, psychiatrist, psychologist or counsellor
9. Someone specialising in children's physical health, such as a hospital or community paediatrician, or occupational therapist
10. Someone from social care, such as a social worker
11. Someone from youth justice, such as a probation officer or someone working in a Youth Offending Team
12. None of these, I am not worried about his / her emotions, behaviour, concentration or difficulties in getting along with people
13. I have not contacted any of these people but I am worried about his / her emotions, behaviour, concentration or difficulties in getting along with people
14. Other – please describe"

New service contact outcome variables coded for the secondary analysis.

New variable	Description	Values of new variable
Informal contact	New categorical binary variable. A yes response to one or more of outcome response categories 1-4.	Yes; no
Professional contact	New categorical binary variable. A yes response to one or more of outcome response categories 5-7 and/or 9-11.	Yes; no

Specialist mental health contact	New categorical binary variable. A yes response to outcome response category 8.	Yes; no
Combined multilevel contact	New categorical nominal variable. Highest level of contact across the four levels.	No contact; informal contact; professional contact; specialist mental health contact

Supplementary Information 2. Data flow through the study.

Total sample: 6718

Age 5-10: 3597 | **Age 11-16:** 3121

↓

Sample with complete outcome data: 6681

Age 5-10: 3583 | **Age 11-16:** 3098

↓

Sample with complete outcome and explanatory variable data: 6457

Age 5-10: 3489 | **Age 11-16:** 2968

The table below shows the frequencies of missing data for each explanatory variable, including “refused", "don't know" or "not applicable" question responses. These question responses were treated as missing data and recoded as such.

Missingness of the dataset

Variable	Missing data - n	Missing data - %*
**Age**	0	0
**Sex**	0	0
**Ethnicity**	5	0.02
**Region**	0	0
**Any physical disorder**	0	0
**Mental health disorder**	0	0
**Two or more stressful life events**	19	0.28
**Special educational needs**	19	0.28
**Ever been excluded or had a managed move between primary schools**	71	1.06
**CYP is a carer**	10	0.14
**Tenure of household**	10	0.13
**Neighbourhood deprivation**	0	0
**HRP marital status**	0	0
**Parent/guardian mental health score by GHQ-12**	75	1.12
**Family functioning score by McMaster FAD**	102	1.52
**Total difficulties score on parent SDQ**	5	0.04

*Percentages reported to 2 decimal places.

Supplementary Information 3. Multivariable analysis.

Several criteria were used to select variables for inclusion in the multivariable models: 1) statistically significant associations between explanatory variables and one or more categories of service contact, 2) non-statistically significant associations but *a priori* evidence of relationships between explanatory variables and service contact, 3) non-statistically significant associations but substantive impacts on the coefficients of other covariates. Two variables had strong prior evidence of association with service contact: mental health status of the CYP and parental rating of mental health difficulties [1]. Thus, these would be included in the models even if non-statistically significant in this sample (criterion 2).

For each age group, a series of multivariable multinomial logistic regression models were produced via an iterative approach, starting with a global model containing all explanatory variables. First, only explanatory variables with statistically significant associations with one or more categories of support/service contact in the global model were retained, using an initial conservative threshold of $p<0.1$. Second, each non-statistically significant explanatory variable was added back into the model individually; if these variables became statistically significant when added back in or they had a substantive impact on the coefficients of other covariates, they were then retained. Finally, only explanatory variables with statistically significant associations at a threshold of $p<0.05$ were retained in the final model. This process is summarised below for each age group separately.

5-10 age group

- Model 1: all explanatory variables (n=14)*
- Model 2: only explanatory variables with statistically significant associations at $p<0.1$ level (n=12).
- Models 3-4: explanatory variables with statistically significant associations at $p<0.1$ level plus each non-statistically significant explanatory variable (n=2) added back in separately. When the non-statistically significant variables were each individually added back into the model, none of them became statistically significant nor did they have a substantive impact on the coefficients of the other covariates. Therefore, these variables were left out of the final model.
- Model 5 – only explanatory variables with statistically significant associations at $p<0.05$ level were retained in the final model (n=10).

*NB. two explanatory variables were not included in the 5-10 age group model building process due to very low observed frequencies in certain outcome groups, such that regression outputs using these variables were nonsensical. These were ‘CYP has caring responsibilities’ and ‘Ever been excluded or had a managed move between primary schools’.

11-16 age group

- Model 1: all explanatory variables (n=16).
- Model 2: only explanatory variables with statistically significant associations at $p<0.1$ level (n=14).

- Models 3-4: explanatory variables with statistically significant associations at $p<0.1$ level plus each non-statistically significant explanatory variable (n=2) added back in separately. When the non-statistically significant variables were each individually added back into the model, none of them became statistically significant nor did they have a substantive impact on the coefficients of the other covariates. Therefore, these variables were left out of the final model.
- Model 5 – only explanatory variables with statistically significant associations at $p<0.05$ level were retained in the final model (n=11).

References:

1. Ford, T., Hamilton, H., Meltzer, H., & Goodman, R. (2008). Predictors of Service Use for Mental Health Problems Among British Schoolchildren. Child and adolescent mental health, 13(1), 32–40. https://doi.org/10.1111/j.1475-3588.2007.00449.x

.

Supplementary Information 4. Supplementary univariable analyses.

Univariable multinomial logistic regression analyses for both the 5-10 and 11-16 age groups were conducted to provide supplementary information for the interested reader.

When comparing the bivariate results below with the final fully adjusted multivariable model results in the main analyses, we see that several predictors lose statistical significance when entered into multivariable models and we also observe changes to the magnitude and statistical significance of the associations of the remaining predictors following adjustment.

Specifically, for the 5-10 age group we find that ‘Sex’, ‘HRP marital status’ and ‘Family functioning score’ are all statistically significant predictors in univariable analyses, but in multivariable analyses these predictors drop out of the model and lose statistical significance at the $p<0.05$ level.

For the 11-16 age group, we find that ‘CYP has caring responsibilities’, ‘Parent/guardian mental health score’ and Family functioning score’ are all statistically significant predictors in univariable analyses, but that in multivariable analyses these predictors drop out of the model and lose statistical significance at the $p<0.05$ level.

Across both age groups, we find that in univariable analyses, living in socially rented accommodation is associated with increased professional and specialist mental health service contact. However, in multivariable analyses we find that the direction of association reverses, with participants living in socially rented accommodation having a lower likelihood of service contact. On further exploration, which comprised adding individual covariates into the multinomial logistic regression model alongside the ‘Household tenure’ variable, we find that it is the addition of the ‘Any mental health disorder’ and ‘Total difficulties score on parent SDQ’ covariates that appear to cause the change in the direction of association in this predictor. These predictors are indicators of underlying mental health needs and thus this finding suggests that, independent of mental health status, CYP living in socially rented accommodation have a lower likelihood of service contact. As we know from the wider literature that socio-economic status is itself often found to be associated with mental health status (with more deprived populations experiencing increased mental health problems), this finding may indicate unmet need.

Univariable multinomial logistic regression analysis of the 5-10 age group

Variable	Informal contact			Professional contact			Specialist mental health contact		
	OR	95% CI	P value	OR	95% CI	P value	OR	95% CI	P value
**Age (years)**	1.06	0.96-1.18	0.250	1.10	1.05-1.16	<0.001	1.43	1.25-1.64	<0.001
**Sex**									
Male (ref.)	-	-	-	-	-	-	-	-	-
Female	0.77	0.54-1.11	0.158	0.66	0.55-0.78	<0.001	0.50	0.32-0.78	0.002
**Ethnicity**									
White (ref.)	-	-	-	-	-	-	-	-	-
BAME	0.54	0.32-0.90	0.017	0.38	0.29-0.49	<0.001	0.32	0.15-0.67	0.002
**Region**	-	-	-	-	-	-	-	-	-

London and South of England (ref.)<br>North of England<br>Midlands and East of England	0.65<br>0.76	0.41-1.01<br>0.50-1.16	0.055<br>0.202	1.08<br>1.11	0.87-1.34<br>0.90-1.37	0.498<br>0.339	1.29<br>1.16	0.77-2.17<br>0.68-1.96	0.336<br>0.583
**Any physical disorder**									
No (ref.)	-	-	-	-	-	-	-	-	-
Yes	1.70	1.18-2.45	0.004	2.06	1.72-2.47	<0.001	2.80	1.76-4.45	<0.001
**Any mental health disorder**									
0 (ref.)	-	-	-	-	-	-	-	-	-
1	3.37	1.96-5.81	<0.001	6.19	4.72-8.11	<0.001	14.51	7.98-26.40	<0.001
≥2	3.66	0.82-16.45	0.090	31.08	17.02-56.75	<0.001	261.21	126.30-540.20	<0.001
**Two or more stressful life events**									
No (ref.)	-	-	-	-	-	-	-	-	-
Yes	1.82	1.15-2.88	0.011	2.52	2.03-3.13	<0.001	3.04	1.88-4.90	<0.001
**Special educational needs**									
No (ref.)	-	-	-	-	-	-	-	-	-
Yes	2.15	1.02-4.54	0.045	8.37	6.24-11.22	<0.001	25.00	15.56-40.19	<0.001
**Ever excluded or had a managed move between primary schools**									
No (ref.)	-	-	-	-	-	-	-	-	-
Yes	*	*	*	*	*	*	*	*	*
**CYP has caring responsibilities**									
No (ref.)	-	-	-	-	-	-	-	-	-
Yes	*	*	*	*	*	*	*	*	*
**Tenure of household**									
Owner occupied (ref.)	-	-	-	-	-	-	-	-	-
Private rented	1.26	0.82-1.93	0.285	1.16	0.93-1.45	0.194	1.35	0.78-2.35	0.284
Social rented	0.73	0.44-1.22	0.236	1.32	1.07-1.64	0.010	1.95	1.19-3.18	0.008
**Neighbourhood deprivation by IMD**									
Least deprived to 3rd quintile (ref.)	-	-	-	-	-	-	-	-	-
4th quintile to most deprived	0.60	0.41-0.87	0.007	0.72	0.61-0.87	<0.001	0.79	0.51-1.22	0.283
**HRP marital status**									
Married or cohabiting (ref.)	-	-	-	-	-	-	-	-	-
Lone parent	0.96	0.60-1.54	0.871	1.71	1.40-2.09	<0.001	1.89	1.18-3.02	0.008
**Parent/guardian mental health score by GHQ-12**									
0-3 (ref.)	-	-	-	-	-	-	-	-	-
≥4	1.32	0.79-2.21	0.286	2.48	1.99-3.09	<0.001	3.30	2.05-5.29	<0.001
**Family functioning**									

**score by McMaster FAD**<br>≤2 (ref.)<br>>2	 -<br>1.63	 -<br>1.04-2.56	 -<br>0.035	 -<br>1.63	 -<br>1.30-2.05	 -<br><0.001	 -<br>2.30	 -<br>1.41-3.75	 -<br>0.001
**Total difficulties score on parent SDQ**	1.12	1.08-1.15	<0.001	1.18	1.16-1.20	<0.001	1.34	1.29-1.38	<0.001

Abbreviations: BAME, Black, Asian and Minority Ethnic; IMD, Index of Multiple Deprivation; HRP, household reference person; GHQ-12, 12-item General Health Questionnaire; FAD, Family Assessment Device; SDQ, Strengths and Difficulties Questionnaire; ref., reference category; OR, Odds Ratio; 95% CI, 95% confidence interval. NB: OR and 95% CI are reported to two decimal places. In all models, 'no contact' was taken as the base outcome. *Outputs from these multinomial regression models were nonsensical as there were cells with frequencies of 0 or 1.

Univariable multinomial logistic regression analysis of 11-16 age group

Variable	Informal contact			Professional contact			Specialist mental health contact		
	OR	95% CI	P value	OR	95% CI	P value	OR	95% CI	P value
**Age (years)**	1.11	1.00-1.24	0.050	0.98	0.92-1.04	0.445	1.04	0.95-1.15	0.399
**Sex**<br>Male (ref.)<br>Female	 -<br>1.24	 -<br>0.86-1.79	 -<br>0.245	 -<br>0.95	 -<br>0.78-1.16	 -<br>0.631	 -<br>1.03	 -<br>0.74-1.44	 -<br>0.860
**Ethnicity**<br>White (ref.)<br>BAME	 -<br>0.57	 -<br>0.35-0.96	 -<br>0.033	 -<br>0.39	 -<br>0.28-0.53	 -<br><0.001	 -<br>0.27	 -<br>0.15-0.51	 -<br><0.001
**Region**<br>London and South of England (ref.)<br>North of England<br>Midlands and East of England	 -<br>1.39<br>1.28	 -<br>0.90-2.16<br>0.81-2.01	 -<br>0.142<br>0.294	 -<br>0.88<br>0.93	 -<br>0.69-1.12<br>0.73-1.18	 -<br>0.301<br>0.551	 -<br>1.40<br>1.27	 -<br>0.94-2.11<br>0.83-1.93	 -<br>0.101<br>0.273
**Any physical disorder**<br>No (ref.)<br>Yes	 -<br>1.55	 -<br>1.07-2.24	 -<br>0.019	 -<br>2.52	 -<br>2.04-3.12	 -<br><0.001	 -<br>1.98	 -<br>1.40-2.81	 -<br><0.001
**Any mental health disorder**<br>0 (ref.)<br>1<br>≥2	 -<br>3.73<br>10.52	 -<br>2.18-6.41<br>4.98-22.20	 -<br><0.001<br><0.001	 -<br>7.25<br>26.81	 -<br>5.39-9.75<br>16.64-43.18	 -<br><0.001<br><0.001	 -<br>21.63<br>135.13	 -<br>13.57-34.47<br>76.38-239.08	 -<br><0.001<br><0.001
**Two or more stressful life events**<br>No (ref.)<br>Yes	 -<br>2.90	 -<br>1.99-4.22	 -<br><0.001	 -<br>2.89	 -<br>2.34-3.58	 -<br><0.001	 -<br>3.72	 -<br>2.64-5.23	 -<br><0.001
**Special educational needs**<br>No (ref.)<br>Yes	 -<br>1.77	 -<br>0.90-3.47	 -<br>0.099	 -<br>5.72	 -<br>4.27-7.65	 -<br><0.001	 -<br>10.06	 -<br>6.78-14.94	 -<br><0.001
**Ever excluded or had a managed move between primary schools**<br>No (ref.)<br>Yes	 -<br>3.02	 -<br>1.46-6.26	 -<br>0.003	 -<br>5.32	 -<br>3.63-7.79	 -<br><0.001	 -<br>6.08	 -<br>3.54-10.44	 -<br><0.001
**CYP has caring responsibilities**<br>No (ref.)	 -	 -	 -	 -	 -	 -	 -	 -	 -

Yes	1.21	0.79-1.85	0.391	1.02	0.80-1.30	0.897	0.89	0.58-1.37	0.592
**Tenure of household**									
Owner occupied (ref.)	-	-	-	-	-	-	-	-	-
Private rented	1.75	1.12-2.76	0.015	1.26	0.95-1.66	0.107	0.89	0.53-1.49	0.654
Social rented	1.12	0.69-1.83	0.645	1.48	1.16-1.89	0.002	1.40	0.94-2.10	0.099
**Neighbourhood deprivation by IMD**									
Least deprived to 3rd quintile (ref.)	-	-	-	-	-	-	-	-	-
4th quintile to most deprived	0.81	0.56-1.17	0.252	0.78	0.64-0.96	0.017	0.83	0.59-1.17	0.287
**HRP marital status**									
Married or cohabiting (ref.)	-	-	-	-	-	-	-	-	-
Lone parent	2.12	1.44-3.11	<0.001	1.61	1.29-2.02	<0.001	2.67	1.89-3.77	<0.001
**Parent/guardian mental health score by GHQ-12**									
0-3 (ref.)	-	-	-	-	-	-	-	-	-
≥4	1.61	1.02-2.56	0.042	1.98	1.55-2.54	<0.001	3.36	2.34-4.84	<0.001
**Family functioning score by McMaster FAD**									
≤2 (ref.)	-	-	-	-	-	-	-	-	-
>2	2.12	1.41-3.19	<0.001	1.63	1.28-2.07	<0.001	2.30	1.59-3.34	<0.001
**Total difficulties score on parent SDQ**	1.11	1.08-1.15	<0.001	1.19	1.17-1.21	<0.001	1.29	1.26-1.33	<0.001

Abbreviations: BAME, Black, Asian and Minority Ethnic; IMD, Index of Multiple Deprivation; HRP, household reference person; GHQ-12, 12-item General Health Questionnaire; FAD, Family Assessment Device; SDQ, Strengths and Difficulties Questionnaire; ref., reference category; OR, Odds Ratio; 95% CI, 95% confidence interval. NB: OR and 95% CI are reported to two decimal places. In all models, ‘no contact’ was taken as the base outcome.

Supplementary Information 5. Additional supplementary univariable analyses in response to reviewer comments.

5a: Investigation of the relationship between total difficulties score on Teacher SDQ and service contact for both the 5-10 and 11-16 age groups.

In the 5-10 age group (n=3489), there were a total of 2009 teacher SDQs completed (58% of sample). Univariable multinomial logistic regression (table below) reveals that in CYP aged 5-10, the relationship between total difficulties score on teacher SDQ and service contact is in-keeping with the findings using the total difficulties score on parent SDQ (see univariable analyses in Supplementary Information section 4 above). Of note, the effect size is slightly lower than that of the relationship between total difficulties score on parent SDQ and service contact.

Univariable multinomial logistic regression analysis of 5-10 age group

Variable	Informal contact			Professional contact			Specialist mental health contact		
	OR	95% CI	P value	OR	95% CI	P value	OR	95% CI	P value
**Total difficulties score on teacher SDQ**	1.08	1.04-1.13	<0.001	1.15	1.13-1.18	<0.001	1.20	1.15-1.25	<0.001

In the 11-16 age group (n=2968), there were a total of 1489 teacher SDQs completed (50% of sample). Univariable multinomial logistic regression (table below) reveals that in CYP aged 11-16, the relationship between total difficulties score on teacher SDQ and service contact is in-keeping with the findings using the total difficulties score on parent SDQ (see univariable analyses in Supplementary Information section 4 above). Of note, the effect size is slightly lower than that of the relationship between total difficulties score on parent SDQ and service contact.

Univariable multinomial logistic regression analysis of 11-16 age group

Variable	Informal contact			Professional contact			Specialist mental health contact		
	OR	95% CI	P value	OR	95% CI	P value	OR	95% CI	P value
**Total difficulties score on teacher SDQ**	1.05	1.00-1.09	0.034	1.14	1.11-1.16	<0.001	1.19	1.15-1.23	<0.001

5b: Investigation of the relationship between total difficulties score on Child SDQ and service contact for the 11-16 age group.

In the 11-16 age group (n=2968), there were a total of 2500 child SDQs completed (84% of sample). Univariable multinomial logistic regression (table below) reveals that in CYP aged 11-16, the relationship between total difficulties score on child SDQ and service contact is in-keeping with the findings using the total difficulties score on parent SDQ (see univariable analyses in Supplementary Information section 4 above). Of note, the effect size is slightly lower than that of the relationship between total difficulties score on parent SDQ and service contact.

Univariable multinomial logistic regression analysis of 11-16 age group

Variable	Informal contact			Professional contact			Specialist mental health contact		
	OR	95% CI	P value	OR	95% CI	P value	OR	95% CI	P value
**Total difficulties score on child SDQ**	1.09	1.05-1.14	<0.001	1.17	1.14-1.19	<0.001	1.20	1.16-1.24	<0.001

Supplementary Information 6. Summary of the evidence base considered to inform explanatory variable selection.

Variable selection was theoretically informed in that potential determinants of service contact were identified through literature review and final decisions on variable inclusion were achieved iteratively through discussion and consensus within the study team. This was done to minimise the total number of explanatory variables, reduce the number of statistical comparisons and thus decrease the likelihood of Type 1 error.

A literature search was conducted using the bibliographic database PubMed to identify the current evidence-base for predictors of mental health service contact in CYP in the UK. The following search strategy was applied using Boolean Operators:

> **(((("service contact") OR ("service use")) AND (("mental health") OR (psychiatric))) AND (((children) OR ("young people")) OR (adolescents))) AND ((((england) OR ("united kingdom")) OR (UK)) OR (Britain))**

The table below summarises characteristics found to be statistically significantly associated with service contact in UK-based studies. The magnitude of the associations varied considerably according to study design, with notable differences in the populations studied and precise classifications of different service contact outcomes. It should also be noted that several of these studies' present analyses of the same or similar datasets, highlighting the paucity of evidence on this topic.

Characteristic	References*
**Age**	Knapp et al., 2015; Shivram et al., 2009
**Sex**	Ford et al., 2008; Knapp et al., 2015; Toms & Totsika, 2015; Young et al., 2011
**Ethnicity**	Durà-Vilà & Hodes, 2009
**Location**	Ford et al., 2008
**Physical disorder**	Ford et al., 2008; Shivram et al., 2009
**Mental disorder**	Ford et al., 2008; Ford et al., 2007; Ford et al., 2005; Potter et al., 2012; Shivram et al., 2009; Toms & Totsika, 2015
**Comorbidity**	Ford et al., 2007; Ford et al., 2005; Potter et al., 2012; Sayal et al., 2010; Shivram et al., 2009
**Recognition and perceived impact of psychopathology**	Ford et al., 2008; Ford et al., 2007; Knapp et al., 2015; Potter et al., 2012; Sayal et al., 2003; Sayal et al., 2010; Sayal et al., 2015
**Stressful life events**	Ford et al., 2008
**Birth complications**	Young et al., 2011
**Bullying**	Evans-Lacko et al., 2017
**Educational status**	Ford et al., 2008; Knapp et al., 2015; Toms & Totsika, 2015

**Excluded from school**	Shivram et al., 2009
**Socioeconomic status**	Knapp et al., 2015
**Family structure**	Ford et al., 2008; Durà-Vilà & Hodes, 2009; Toms & Totsika, 2015; Young et al., 2011
**Tenure of household**	Ford et al., 2008
**Ever been looked after by local authority**	Shivram et al., 2009
**Parental marital status**	Ford et al., 2008; Shivram et al., 2009; Young et al., 2011
**Parental mental health**	Ford et al., 2008; Knapp et al., 2015; Potter et al., 2012; Sayal et al., 2010; Sayal et al., 2015; Toms & Totsika, 2015
**Family functioning**	Knapp et al., 2015

*Full references can be found listed below

Durà-Vilà, G., & Hodes, M. (2009). Ethnic variation in service utilisation among children with intellectual disability. Journal of intellectual disability research : JIDR, 53(11), 939–948. https://doi.org/10.1111/j.1365-2788.2009.01214.x

Evans-Lacko, S., Takizawa, R., Brimblecombe, N., King, D., Knapp, M., Maughan, B., & Arseneault, L. (2017). Childhood bullying victimization is associated with use of mental health services over five decades: a longitudinal nationally representative cohort study. Psychological medicine, 47(1), 127–135. https://doi.org/10.1017/S0033291716001719

Ford, T., Hamilton, H., Meltzer, H., & Goodman, R. (2008). Predictors of Service Use for Mental Health Problems Among British Schoolchildren. Child and adolescent mental health, 13(1), 32–40. https://doi.org/10.1111/j.1475-3588.2007.00449.x

Ford, T., Hamilton, H., Meltzer, H., & Goodman, R. (2007). Child Mental Health is Everybody's Business: The Prevalence of Contact with Public Sector Services by Type of Disorder Among British School Children in a Three-Year Period. Child and adolescent mental health, 12(1), 13–20. https://doi.org/10.1111/j.1475-3588.2006.00414.x

Ford, T., Hamilton, H., Goodman, R., & Meltzer, H. (2005). Service Contacts Among the Children Participating in the British Child and Adolescent Mental Health Surveys. Child and adolescent mental health, 10(1), 2–9. https://doi.org/10.1111/j.1475-3588.2005.00108.x

Knapp, M., Snell, T., Healey, A., Guglani, S., Evans-Lacko, S., Fernandez, J. L., Meltzer, H., & Ford, T. (2015). How do child and adolescent mental health problems influence public sector costs? Interindividual variations in a nationally representative British sample. Journal of child psychology and psychiatry, and allied disciplines, 56(6), 667–676. https://doi.org/10.1111/jcpp.12327

Potter, R., Mars, B., Eyre, O., Legge, S., Ford, T., Sellers, R., Craddock, N., Rice, F., Collishaw, S., Thapar, A., & Thapar, A. K. (2012). Missed opportunities: mental disorder in children of parents with depression. The British journal of general practice : the journal of the Royal College of General Practitioners, 62(600), e487–e493. https://doi.org/10.3399/bjgp12X652355

Sayal, K., Taylor, E., & Beecham, J. (2003). Parental perception of problems and mental health service use for hyperactivity. Journal of the American Academy of Child and Adolescent Psychiatry, 42(12), 1410–1414. https://doi.org/10.1097/00004583-200312000-00007

Sayal, K., Ford, T., & Goodman, R. (2010). Trends in recognition of and service use for attention-deficit hyperactivity disorder in Britain, 1999-2004. Psychiatric services (Washington, D.C.), 61(8), 803–810. https://doi.org/10.1176/ps.2010.61.8.803

Sayal, K., Mills, J., White, K., Merrell, C., & Tymms, P. (2015). Predictors of and barriers to service use for children at risk of ADHD: longitudinal study. European child & adolescent psychiatry, 24(5), 545–552. https://doi.org/10.1007/s00787-014-0606-z

Shivram, R., Bankart, J., Meltzer, H., Ford, T., Vostanis, P., & Goodman, R. (2009). Service utilization by children with conduct disorders: findings from the 2004 Great Britain child mental health survey. European child & adolescent psychiatry, 18(9), 555–563. https://doi.org/10.1007/s00787-009-0012-0

Toms, G., Totsika, V., Hastings, R., & Healy, H. (2015). Access to services by children with intellectual disability and mental health problems: Population-based evidence from the UK. Journal of Intellectual & Developmental Disability, 40(3), 239–247. https://doi.org/10.3109/13668250.2015.1045460

Young, R., Riordan, V., & Stark, C. (2011). Perinatal and psychosocial circumstances associated with risk of attempted suicide, non-suicidal self-injury and psychiatric service use. A longitudinal study of young people. BMC public health, 11, 875. https://doi.org/10.1186/1471-2458-11-875
